# A concise review of the regulatory, diagnostic, and prognostic implications of HOXB-AS3 in tumors

**DOI:** 10.7150/jca.91033

**Published:** 2024-01-01

**Authors:** Hongze Wu, Jiarong Ye, Mengqi Zhang, Hongliang Luo

**Affiliations:** 1 Department of Gastrointestinal Surgery, The Second Affiliated Hospital of Nanchang University, Nanchang 330000, Jiangxi, China.; 2 Department of Traditional Chinese Medicine, Jiujiang Hospital of Traditional Chinese Medicine, Jiujiang 332007, Jiangxi, China.; 3 Nanchang University Queen Mary School, Nanchang 330038, Jiangxi, China.; 4 The Second Clinical Medical College, Nanchang University, Nanchang 330038, Jiangxi, China.

**Keywords:** HOXB-AS3, human tumors, clinical significance, molecular target, biological functions, regulatory mechanisms

## Abstract

Recent studies have reported that HOXB-AS3 (HOXB Cluster Antisense RNA 3) is an intriguing molecule with dual functionality as a long noncoding RNA (lncRNA) and putative coding peptide in tumorigenesis and progression. The significant expression alterations of HOXB-AS3 were detected in diverse cancer types and closely correlated with clinical stage and patient survival. Furthermore, HOXB-AS3 was involved in a spectrum of biological processes in solid tumors and hematological malignancies, such as stemness, lipid metabolism, migration, invasion, and tumor growth. This review comprehensively analyzes its clinical relevance for diagnosis and prognosis across human tumors and summarizes its functional role and regulatory mechanisms in different malignant tumors, including liver cancer, acute myeloid leukemia, ovarian cancer, lung cancer, endometrial carcinoma, colon cancer, and oral squamous cell carcinoma. Overall, HOXB-AS3 emerges as a promising biomarker and novel therapeutic target in multiple human tumors.

## Introduction

Cancer, a leading cause of global mortality, continues to be a significant public health concern. According to recent data, in 2020, there were approximately 19.3 million new cancer cases and 10 million deaths worldwide 1, 2. This represents a substantial increase from the reported figures in 1990. In China, there were an estimated 4.82 million new cancer cases and 3.21 million cancer-related deaths in 2022 3. Similarly, in the United States, it is projected that there were around 1.92 million new cancer cases and 0.61 million deaths in 2022 4. The rising incidence and mortality rates of cancer highlight the urgent need for innovative approaches in diagnosis, prognosis, and treatment.

Extensive research efforts have been dedicated to the identification of biomarkers and molecular targets for cancer therapy 5-9. Among these, the exploration of long non-coding RNAs (lncRNAs) has garnered significant attention 10, 11. LncRNAs are a type of RNA with a length exceeding 200 nucleotides that do not encode proteins or have limited protein-coding ability 12. They can be broadly categorized as sense, antisense, intronic, bidirectional, or intergenic, depending on their relationship with neighboring protein-coding genes 12, 13. In recent years, lncRNAs have gained increasing importance in cancer research, as considerable evidence 14-18 suggests their involvement in tumor initiation and progression by influencing various cellular processes, including cell cycle, survival, tumor invasion, and metastasis through multiple mechanisms at the transcriptional, post-transcriptional, or epigenetic levels. Importantly, lncRNAs have demonstrated promise as potential diagnostic and prognostic markers, as well as therapeutic targets in various types of cancer 19-22.

**Figure 1:**
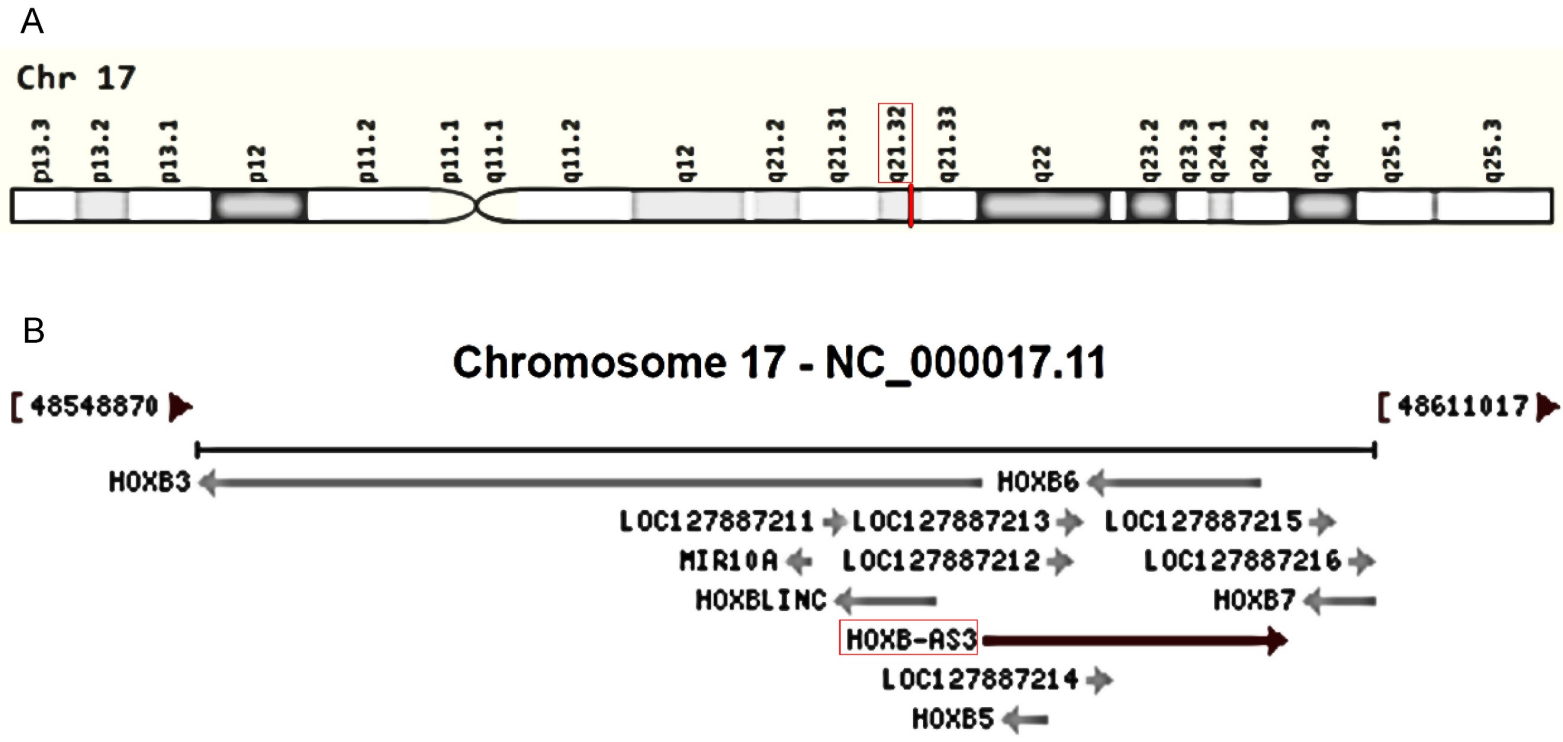
Genomic view for HOXB-AS3 gene for genomic location (A) extracted from GeneCards database (https://www.genecards.org/cgi-bin/carddisp.pl?gene=HOXB-AS3&keywords=HOXB-AS3), and genomic context (B) from NCBI database (https://www.ncbi.nlm.nih.gov/gene/404266).

HOX genes, encompassing the HOXA, HOXB, HOXC, and HOXD gene clusters, play a pivotal role in tumorigenesis and serve as valuable drug targets in disease treatment 23-26. Recently, there has been an increasing focus on lncRNAs associated with these genes in various aspects of cancer, including HOXA-AS3 27-31, HOTTIP 32-35, and HOTAIR 36-39. Among them, HOXB-AS3 (HOXB Cluster Antisense RNA 3) has emerged as a novel antisense lncRNA transcribed from the human chromosome 17q21.32 (**Figure 1A**), overlapping with the HOXB5 and HOXB6 genes on the sense strands (**Figure 1B**). Since its discovery, HOXB-AS3 has been implicated in cancers. Initially, HOXB-AS3, which is annotated as a lncRNA, was found actually encodes a small peptide that is reported to participate in suppressing colon cancer growth 40, however, subsequent studies primarily focused on the RNA level and revealed tumor-promoting properties of HOXB-AS3 RNA 41-47. For instance, in liver cancer 47, lncRNA HOXB-AS3 is significantly upregulated in cancerous samples, particularly in the advanced stage. This upregulation enhances tumor cell proliferation while inhibiting apoptosis. Similarly, in endometrial carcinoma (EC) 42, both cancer tissues and EC cell lines exhibit overexpression of lncRNA HOXB-AS3, and lncRNA HOXB-AS3 correlates with survival rates in EC patients. HOXB-AS3 overexpression could promote EC progression 42. Furthermore, research also unveiled the regulatory role of HOXB-AS3 on tumor development, involving multiple pathways 43, 44, 47, 48. HOXB-AS3 demonstrates promising potential as an oncological biomarker and therapeutic target in human malignant disorders.

In this review, we systematically searched PubMed and Google Scholar using terms related to HOXB-AS3 and manually screened relevant full-text studies of human malignancies. By synthesizing the results of these studies and combining multiple online databases, we systematically analyzed the aberrant expression of HOXB-AS3 and its clinical relevance in the prognostic and diagnostic value in different human tumors and also outlined the current understanding of the biological functions and regulatory mechanisms of the HOXB-AS3 lncRNA and its encoded micro-peptides in multiple malignant tumors.

## Promising Clinical Biomarker in Human Cancer

Recent research studies have shown the aberrant expression of HOXB-AS3 in tumor tissues and cancer cell lines, and significant correlations have been established between the dysregulation of HOXB-AS3 and specific clinicopathological characteristics, along with the prognosis of patients across multiple types of tumors, as presented in **Table 1**. In this section, we aim to provide a comprehensive overview and investigation into the changes observed in the expression of HOXB-AS3 across human malignancies. Additionally, we explore the relationship between HOXB-AS3 and patient prognoses, such as overall survival (OS), disease-specific survival (DSS), and progression-free interval (PFI), and discuss its potential as a valuable diagnostic biomarker for different tumor types.

### Gene Expression Across Human Cancers

In recent studies, aberrant expression of the HOXB-AS3 RNA has been found in liver cancer 47, 49, lung cancer 43, ovarian cancer 41, 44, endometrial cancer 42, 48, 51, and acute myeloid leukemia 45, 46, 50. And at the protein expression level, abnormal expression of the HOXB-AS3 micro-peptide has been reported in oral squamous cell carcinoma 52 and colon cancer 40 (**Table 1**).

**Figure 2:**
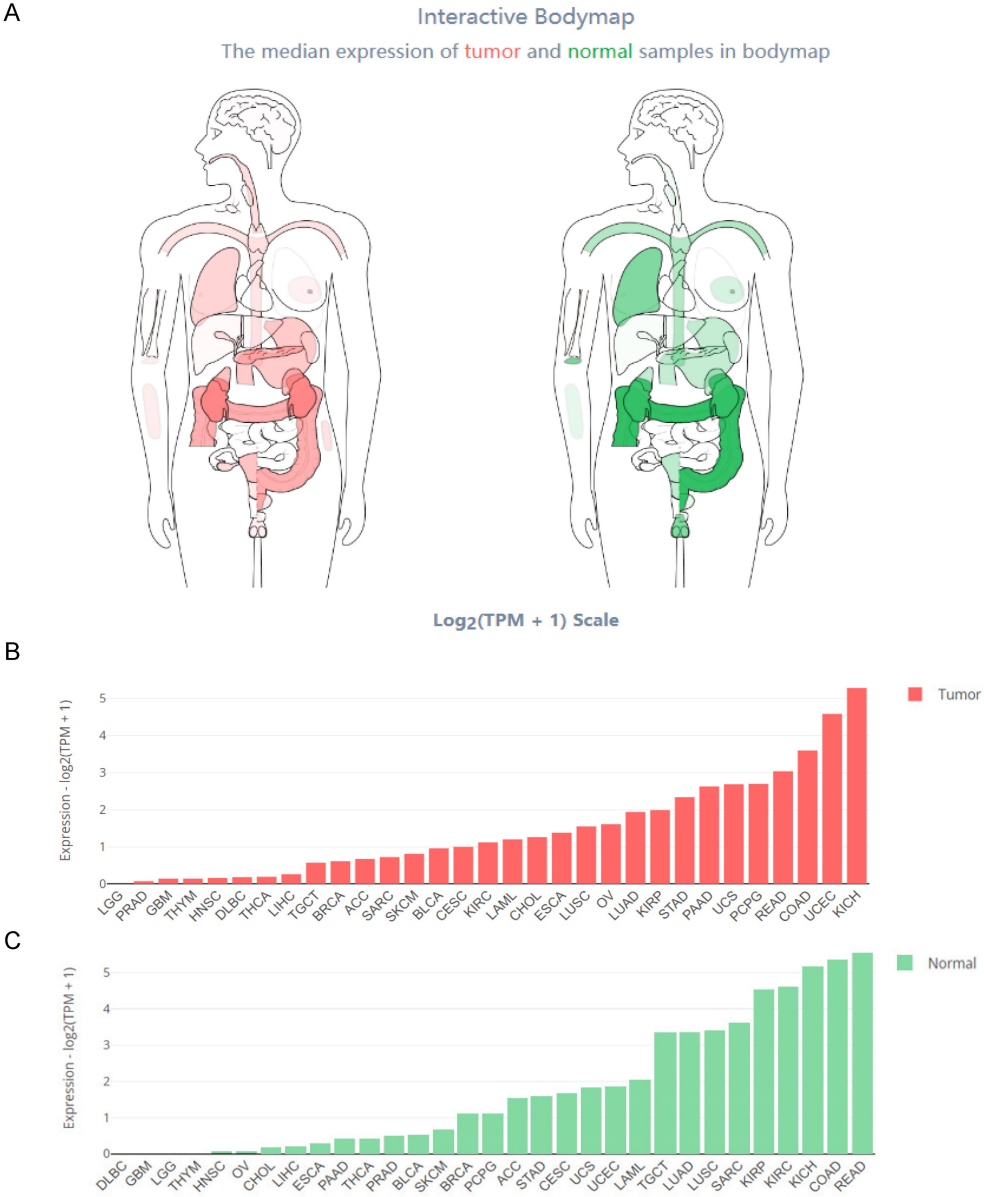
Comprehensive Expression Profile of HOXB-AS3 Isoforms Across Human Tissues. Analysis of HOXB-AS3 gene expression encompassing all identified isoforms, utilizing data from GEPIA 2: Interactive Bodymap delineates the median expression of HOXB-AS3 in tumor and normal samples (A). Bar plots provide a detailed gene expression profile across tumor samples (B) and corresponding normal tissues (C). The acronyms used in this figure are listed in Supplementary Table 1 for reference.

**Figure 3:**
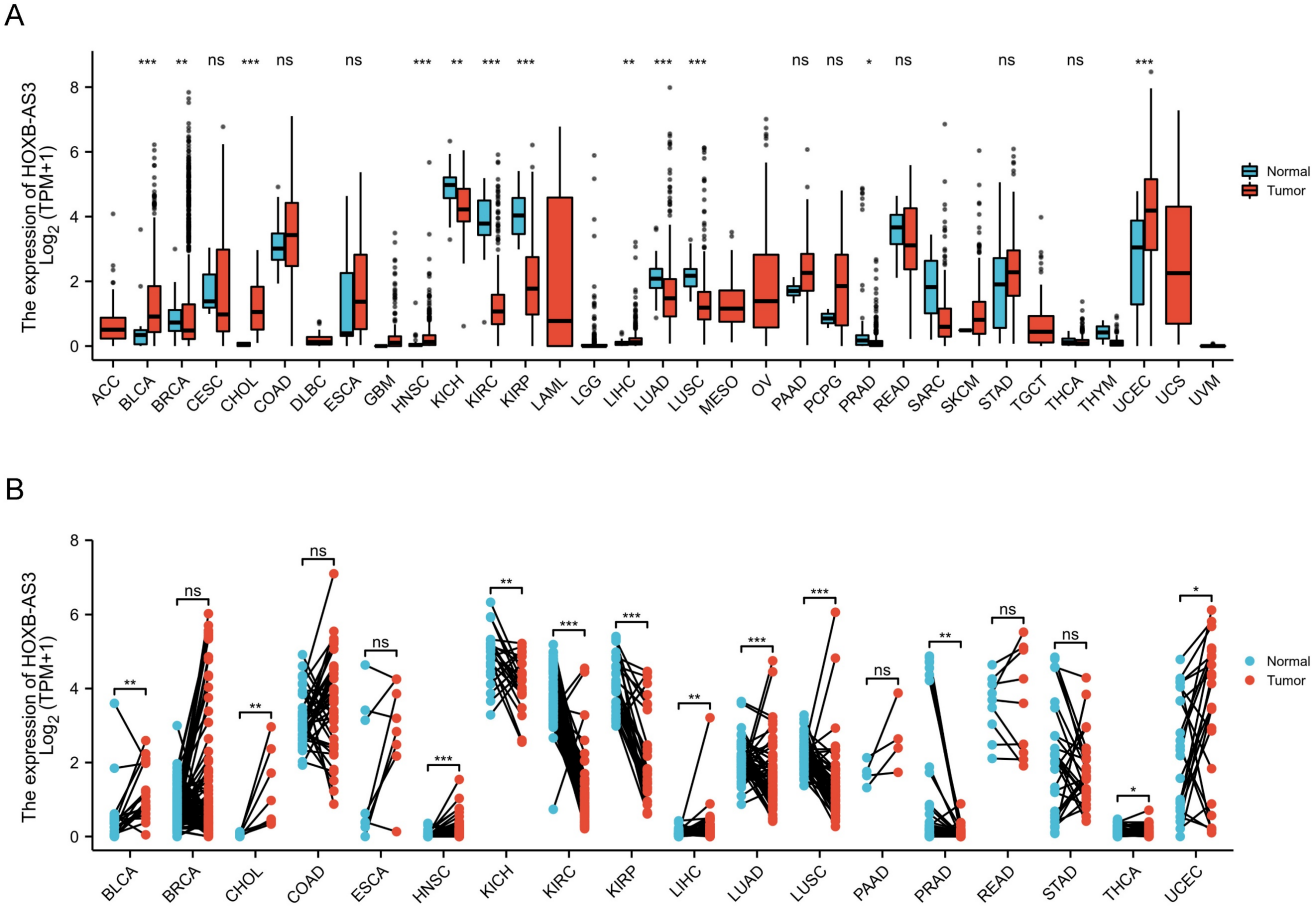
The relative expression level of HOXB-AS3 assessed in pan-cancers (A), as well as in paired normal and tumor samples (B). The acronyms used in this figure are listed in Supplementary Table 1 for reference.

The Interactive Bodymap in GEPIA 2 (http://gepia2.cancer-pku.cn/) displayed the expression profile of the HOXB-AS3 RNA transcript across tumor and normal tissues (**Figure 2A**). Among the tumor samples, KICH exhibited the highest RNA transcript expression (**Figure 2B**), while among the paired normal tissues, the normal tissues adjacent to READ showed the highest RNA transcript expression (**Figure 2C**). To compare the expression levels between tumor and normal samples, the HOXB-AS3 expression levels were assessed in pan-cancers, as well as in paired normal and tumor samples, using UCSC XENA (https://xenabrowser.net/datapages/) (**Figure 3A and B**). The results demonstrated that HOXB-AS3 was significantly up-regulated in a number of malignancies, such as BLCA, CHOL, HNSC, LIHC, and UCEC. Conversely, it was noticeably downregulated in several types of tumors, such as KICH, KIRC, KIRP, and PRAD. The differential expression patterns observed across various cancer types suggest that HOXB-AS3 expression levels may hold significant clinical value in predicting disease onset and progression.

### Prognostic and Diagnostic Potential

The clinical value of HOXB-AS3 as a predictive biomarker has been reported in multiple studies (**Table 1**). HOXB-AS3 expression levels have been found to correlate with clinical features across several cancers (**Table 1**). For instance, in epithelial ovarian cancer, overexpression of HOXB-AS3 RNA was distinctly associated with higher histological grade, advanced FIGO stage and lymph node metastasis 44. Similarly, in lung cancer, the expression level of HOXB-AS3 RNA in tissues was closely linked to tumor differentiation and TNM stage 43. Furthermore, investigations have explored the relationship between HOXB-AS3 expression and patient prognosis in various tumors (**Table 1**). In acute myeloid leukemia 46, epithelial ovarian cancer 44, endometrial carcinoma 42, and oral squamous cell carcinoma 52, higher expression of HOXB-AS3 RNA indicated a poorer prognosis. Conversely, in colon cancer, higher expression of the HOXB-AS3 micro‑peptide was associated with longer survival time 40.

**Figure 4:**
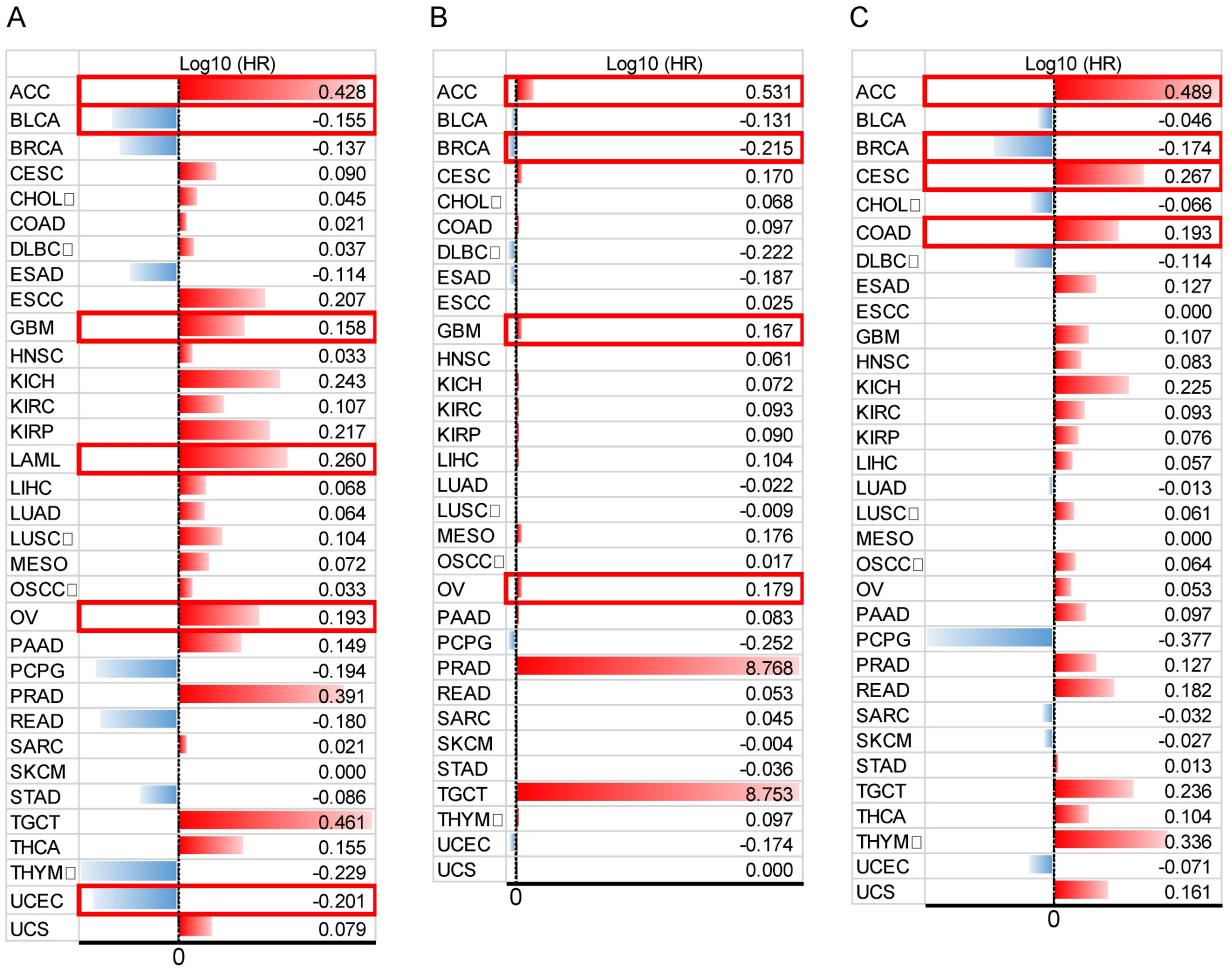
Data bars showing overall survival (OS) (A), disease-specific survival (DSS) (B), and progression-free interval (PFI) (C) in pan-cancer for HOXB-AS3. The horizontal bars represent the data, while the corresponding log10 (hazard ratio) (HR) values are presented on the far right. The cut-off value was determined using the median of HOXB-AS3 expression. Statistical significance was established at a p-value of < 0.05 and is marked with a red outer box. The acronyms used in this figure are listed in Supplementary Table 1 for reference.

In addition, to comprehensively evaluate the prognostic significance of HOXB-AS3 in pan-cancer, we conducted an extensive analysis using publicly available datasets from The Cancer Genome Atlas (TCGA) (https://portal.gdc.cancer.gov/) (**Figure 4A - C**). Regarding OS (**Figure 4A**), we observed that HOXB-AS3 overexpression was associated with an unfavorable prognosis in ACC, GBM, LAML, and OV. Conversely, it acted as a favorable predictor in BLCA and UCEC. In terms of DSS (**Figure 4B**), high expression of HOXB-AS3 was linked to an unfavorable outcome in ACC, GBM, and OV, while it served as a favorable predictor in BRCA.

**Figure 5:**
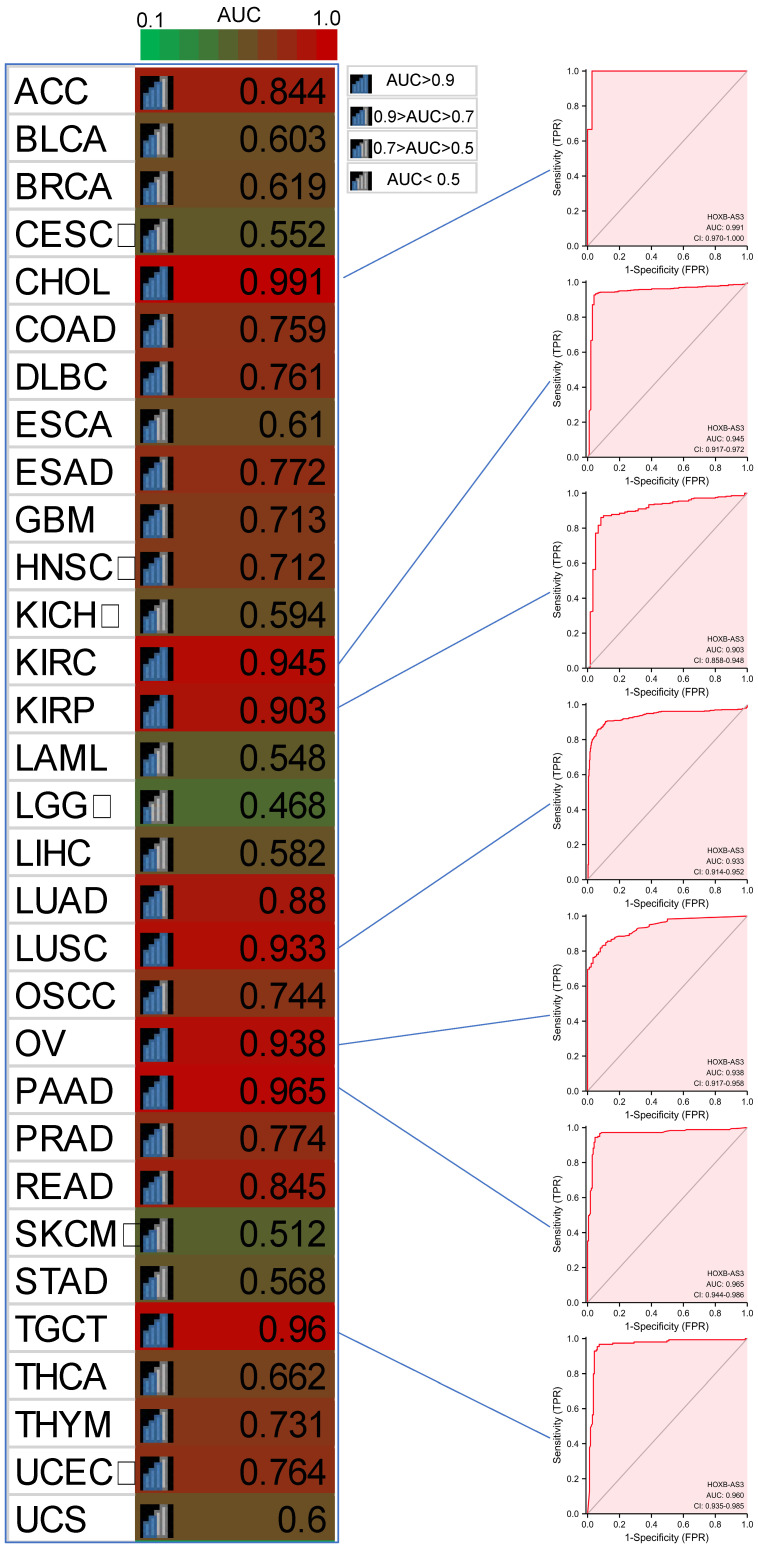
Diagnostic ROC curves for HOXB-AS3 expression in normal and cancer tissues using UCSC XENA datasets. The acronyms used in this figure are listed in Supplementary Table 1 for reference.

Furthermore, in PFI analysis (**Figure 4C**), HOXB-AS3 overexpression was indicative of an unfavorable prognosis in ACC, CESC, and COAD. Conversely, it acted as a favorable predictive factor in BRCA. Furthermore, receiver operating characteristic (ROC) curve analyses were also performed (**Figure 5**), and interestingly, it revealed that HOXB-AS3 expression could serve as a potent diagnostic biomarker in multiple cancer types, particularly in CHOL, KIRC, KIRP, LUSC, OV, PAAD, and TGCT with an area under the curve (AUC) exceeding 0.9. All above results indicated that HOXB-AS3 holds promise as both a diagnostic and prognostic predictor in diverse malignancies.

## Role and Mechanisms of HOXB-AS3 in Cancer

**Figure 6:**
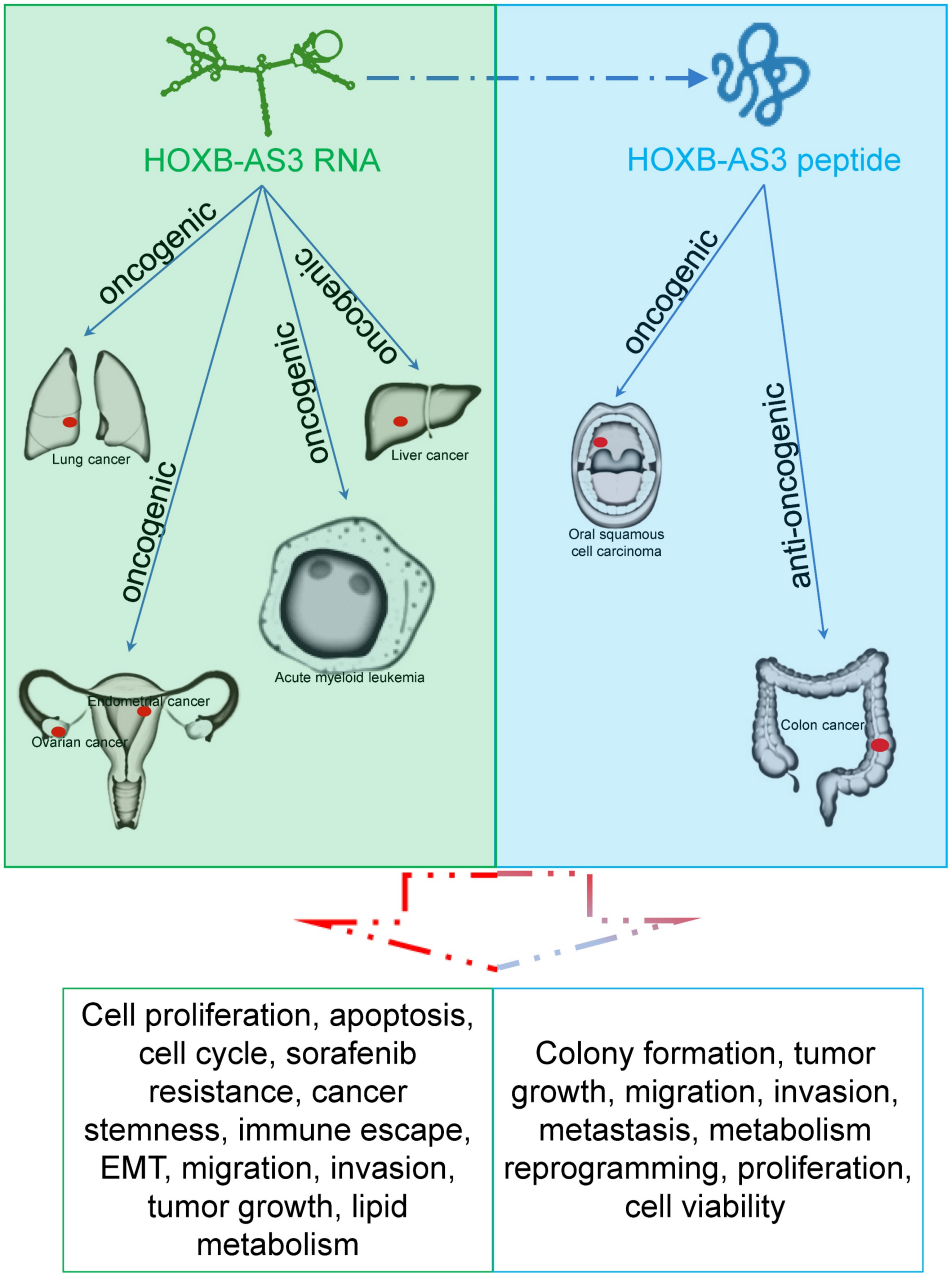
The main role of HOXB-AS3 RNA and its encoded micro-peptide in the occurrence and development of multiple tumors.

HOXB-AS3 RNA and its encoded micro-peptide have both been reported to play crucial roles in regulating a wide range of biological functions that impact tumor initiation and progression. These functions include cell proliferation, viability, apoptosis, cell cycle, migration, invasion, metastasis, tumor growth, epithelial-mesenchymal transition (EMT), stemness, immune escape, and lipid metabolic reprogramming (**Table 2 and Figure 6**). Considering these findings, HOXB-AS3 holds promise as a novel therapeutic target for human cancers.

### HOXB-AS3 lncRNA: The Noncoding Dimension

**Figure 7:**
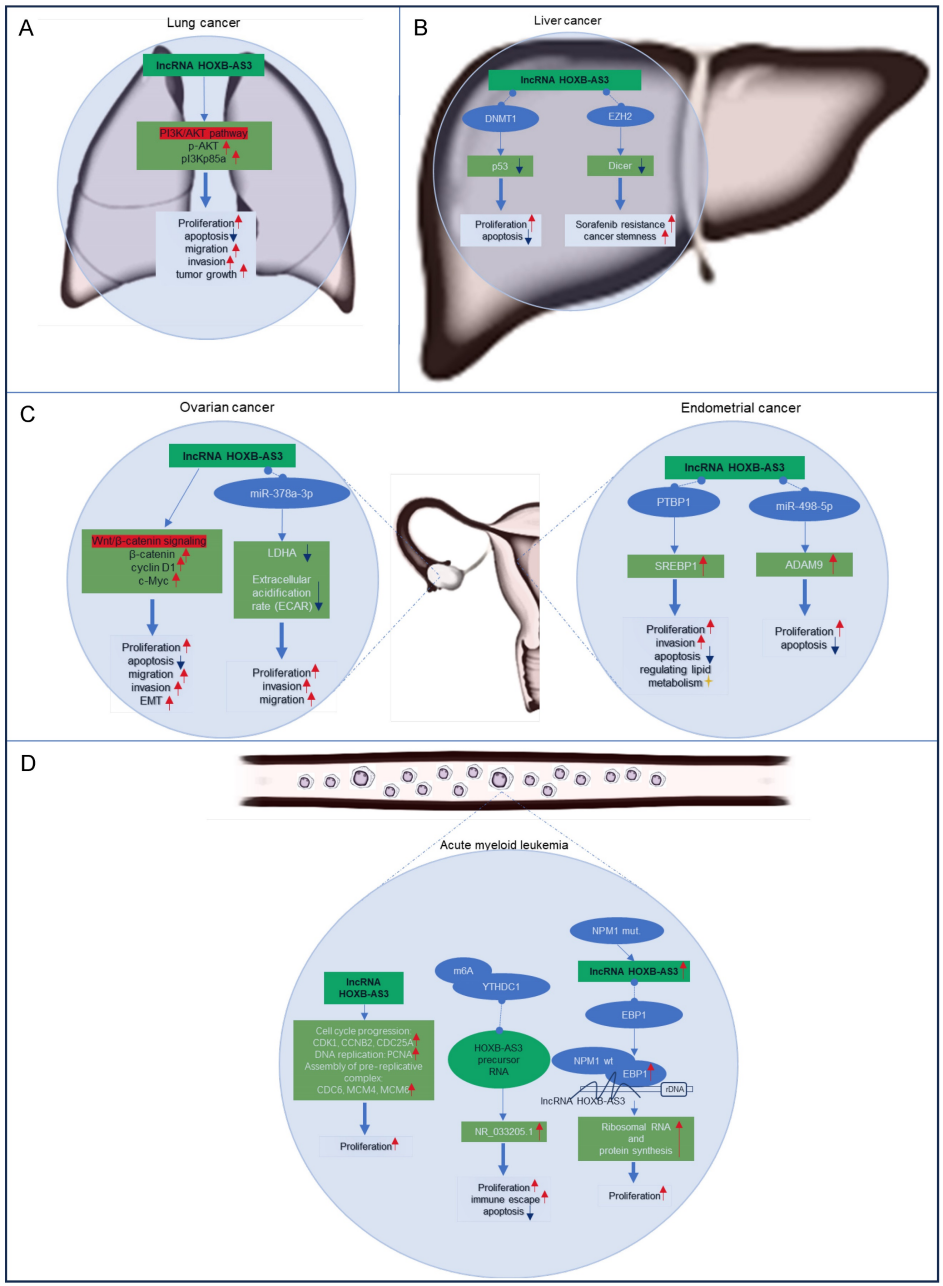
Regulatory mechanisms of lncRNA HOXB‑AS3 in lung cancer (A), liver cancer (B), ovarian cancer and endometrial carcinoma (C), and acute myeloid leukemia (D).

Currently most studies have focused on the functional significance of lncRNA HOXB-AS3 across various cancer types, encompassing lung cancer 43, liver cancer 47, 49, ovarian cancer 41, 44, endometrial cancer 42, 48, 51, and acute myeloid leukemia 45, 46, 50. The regulatory mechanisms of lncRNA HOXB-AS3 in these tumors are delineated in **Figure 7**. This section aims to provide an overview of the distinct regulatory mechanisms attributed to lncRNA HOXB-AS3 in different tumor contexts.

#### Lung Cancer

Lung cancer ranks as the primary cause of tumor-related mortality globally. Increased expression of HOXB-AS3 RNA has been observed in lung cancer tissues and cell lines, exhibiting a noteworthy association with tumor differentiation and TNM stage 43. Functionally, *in vitro* investigations have revealed that inhibition of HOXB-AS3 leads to suppressed cell proliferation, colony formation, induced apoptosis, and reduced migration and invasion capabilities 43. Additionally, *in vivo* experiments have validated the involvement of HOXB-AS3 in promoting tumor growth through the activation of the PI3K-AKT pathway 43. These findings highlight the potential of targeting HOXB-AS3 as a promising therapeutic approach for lung cancer.

#### Liver Cancer

Liver cancer is a highly prevalent and aggressive malignancy. The expression of HOXB-AS3 is significantly upregulated in liver cancer tissues compared to adjacent normal tissues, showing a positive correlation with tumor stage 47. Functional studies have revealed that interfering with HOXB-AS3 expression affects hepatoma cell proliferation, apoptosis, cancer stemness, and sorafenib resistance 47, 49. Mechanistically, HOXB-AS3 binds to DNMT1, leading to the downregulation of the tumor suppressor gene p53 and promoting tumor cell proliferation 47. Additionally, HOXB-AS3 epigenetically suppresses dicer expression by recruiting EZH2, resulting in stem-like cell properties and increased resistance to sorafenib 49. These findings stressed the role of HOXB-AS3 in hepatocellular carcinoma progression and its potential as a therapeutic target for improving treatment outcomes.

#### Ovarian and Endometrial Carcinomas

Ovarian cancer and endometrial carcinoma are the two most common gynecologic tumors, and the elevated expression of lncRNA HOXB-AS3 has been observed in both cancer specimens and cancer cell lines associated with these two cancers 41, 42, 44, 48. In ovarian cancer, elevated levels of HOXB-AS3 indicated an adverse prognosis 41, 44, and HOXB-AS3 overexpression is linked to higher histological grade, advanced FIGO stage, and lymph node metastasis in patients 44. And HOXB-AS3 plays a critical role in ovarian cancer tumorigenesis by activating the Wnt/β-catenin signaling pathway 44 or acting as a miR-378a-3p sponge 41. These molecular mechanisms impact essential cellular processes, including proliferation, apoptosis, migration, invasion, and EMT, underscoring its oncogenic role in epithelial ovarian cancer progression 41, 44. Similarly, in endometrial carcinoma, HOXB-AS3 has been shown to play an oncogenic role 42, 48, 51. The lncRNA HOXB-AS3 serves as a sponge for miR-498-5p or interacts with PTBP1 protein to regulate downstream target genes, which further regulate lipid metabolism and tumor-related biological behaviors, such as cell proliferation, apoptosis, and invasion, to promote tumor progression 42, 48.

#### Acute Myeloid Leukemia

Acute myeloid leukemia (AML) is the predominant form of acute leukemia in adults 53, 54, characterized by the uncontrolled proliferation of abnormal and immature cells in the blood system, disrupting the normal production of blood cells.

In AML, an upregulation of HOXB-AS3 expression has been observed specifically in leukemic stem cells (LSCs), which play a crucial role in leukemogenesis 55-57. Patients with elevated levels of HOXB-AS3 expression have shown a significant decrease in overall survival and relapse-free survival rates compared to those with lower expression levels 46. HOXB-AS3 is implicated in promoting enhanced cell proliferation by upregulating key genes involved in cell cycle progression, DNA replication, and pre-replicative complex assembly 46. Notably, a distinctive mechanism involves the overexpression of the spliceosome NR_033205.1 of HOXB-AS3, facilitated by the RNA-binding protein YTHDC1 through m6A modification of the HOXB-AS3 precursor RNA 50. This aberrant overexpression contributes to proliferation, inhibits apoptosis, and promotes self-renewal of LSCs, exacerbating the leukemic phenotype 50.

In the context of AML cases harboring nucleophosmin 1 (NPM1) mutations 45, HOXB-AS3 assumes a regulatory role in the proliferative capacity of blasts, which are undifferentiated leukemia cells. It accomplishes this through its interaction with ErbB3-binding protein 1 (EBP1), guiding EBP1 to the ribosomal DNA locus 45. This intricate mechanism modulates ribosomal RNA transcription and de novo protein synthesis, pivotal processes for the maintenance of cellular functions and malignant growth in AML.

### HOXB-AS3 Beyond RNA: The Micro-Peptide Dimension

**Figure 8:**
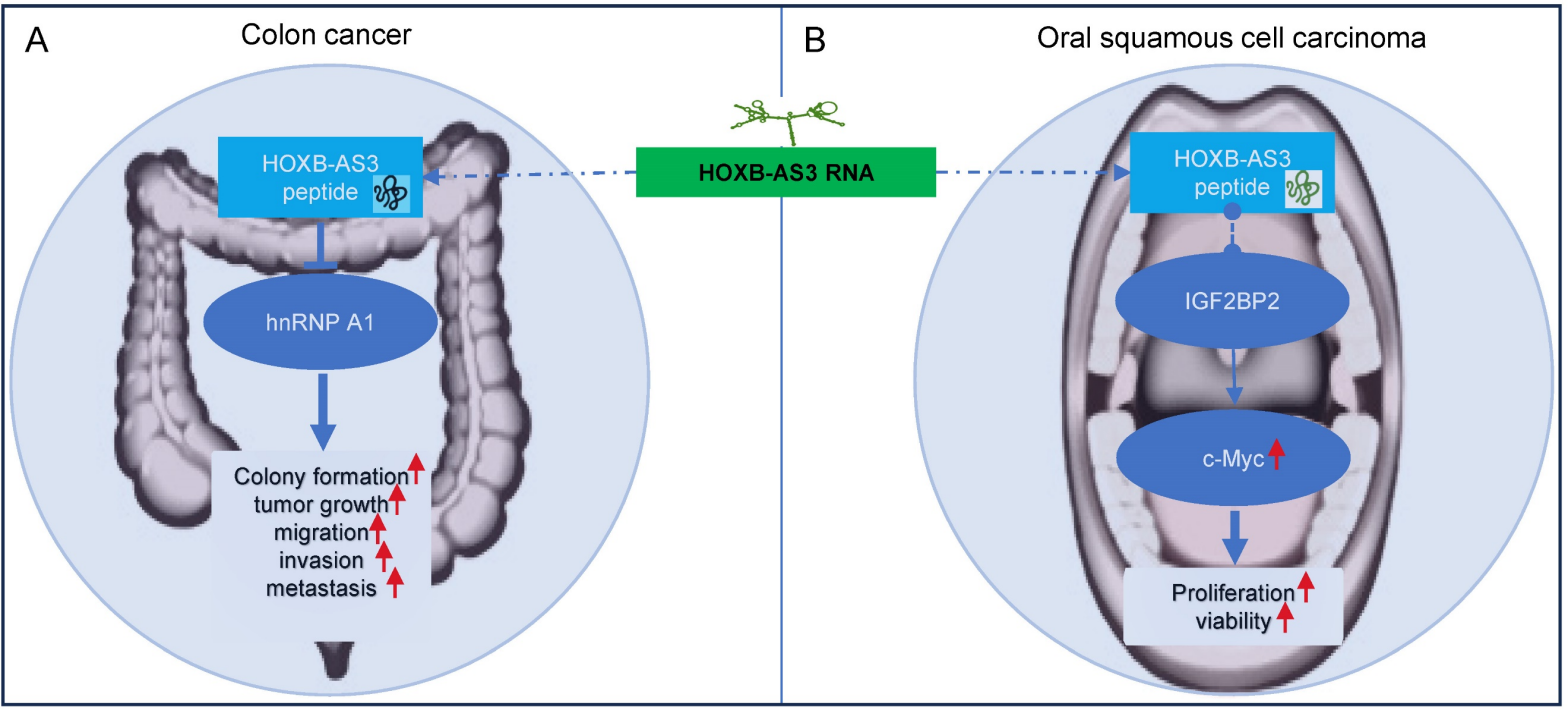
Regulatory mechanisms of HOXB‑AS3 micro-peptide in colon cancer (A) and oral squamous cell carcinoma (B).

HOXB-AS3 was initially identified as a lncRNA gene in Homo sapiens. However, ribosome profiling studies have revealed that HOXB-AS3 RNA associates with ribosomes 58, hinting at its potential to encode a protein or a small peptide. An open reading frame (ORF) within HOXB-AS3, conserved across primates, has been shown to encode a 53-amino acid micro-peptide absent in other species and lacking known protein homology, indicating a unique functional entity encoded by this lncRNA. Huang et al. 40 confirmed that the presence of this HOXB-AS3 micro-peptide in various cancer cell lines, including colon, breast, ovarian, and nasopharyngeal cancer cells. It's noteworthy that the dysregulated expression of the HOXB-AS3 micro-peptide, rather than the lncRNA itself, has been reported to play a crucial role in modulating tumor growth and cell proliferation in colon and oral cancers 40, 52 (**Figure 8**).

Experimental validations have further solidified the coding potential of HOXB-AS3 40. Constructs designed to express HOXB-AS3 ORF (Open Reading Frame) fusion proteins with GFP and Flag tags confirmed peptide expression, dependent on the intact start codon of the HOXB-AS3 ORF. Western blot analyses using anti-GFP antibodies substantiated the expression of these fusion proteins in transfected cells.

The specificity of the peptide's expression was corroborated by the development of antibodies against the HOXB-AS3 peptide itself, which detected the peptide in cancer cell lines, thus underscoring its endogenous presence 40. Most compellingly, the use of a translation-blocking antisense oligonucleotide specific to HOXB-AS3, followed by mass spectrometry, confirmed the peptide's independent translation from the HOXB-AS3 RNA 40, decisively establishing its existence beyond theoretical prediction. These findings necessitate a re-evaluation of HOXB-AS3's role in cancer biology, suggesting a novel functional dimension beyond its RNA origins.

Gaining a comprehensive understanding of the role and regulatory mechanisms of the HOXB-AS3 micro-peptide holds promise for developing effective treatment strategies and exploring its potential clinical applications. This multi-dimensional approach could pave the way for novel therapeutic and diagnostic avenues, harnessing the hitherto unexplored coding capacity of lncRNAs.

#### Colon Cancer and Oral Squamous Cell Carcinoma

Colon cancer and oral squamous cell carcinoma (OSCC) are prevalent cancers with significant global impact 2, 59. They rank as the 3rd and 12th most common malignancies worldwide, respectively 2, 60. In colorectal cancer (CRC), approximately 60% of patients are affected by metastasis, resulting in poor prognoses characterized by five-year survival rates below satisfactory levels 61, 62. Similarly, OSCC presents a concerning scenario, with an overall five-year survival rate in 2020 falling below 50% 63 52.

In colon cancer 40, the HOXB-AS3 micro-peptide displays aberrant expression patterns, with down-regulated levels observed in tumor tissues compared to adjacent normal tissues. Low levels of HOXB-AS3 peptide are associated with a poor prognosis for CRC patients 40. Notably, it is the HOXB-AS3 micro-peptide, rather than the RNA, that acts as a tumor suppressor, exerting functional roles in critical cellular processes like cell proliferation, migration, and invasion, thereby inhibiting tumor growth and metastasis in colon cancer 40. Mechanistically, the HOXB-AS3 peptide suppresses CRC growth by inhibiting hnRNP A1-mediated PKM splicing, consequently preventing PKM2 formation and reprogramming of glucose metabolism 40.

In OSCC 52, the HOXB-AS3 micro-peptide has been reported to exhibit increased expression in tumor tissues. Interestingly, the HOXB-AS3 protein, not the mRNA, exerts its oncogenic function in OSCC tumorigenesis 52. It primarily influences cell proliferation and viability. Regulatory mechanisms involving the HOXB-AS3 micro-peptide in OSCC include its direct binding with IGF2BP2, which enhances the stability of c-Myc mRNA, and leads to increased expression of c-Myc 52. The c-myc transcription factor is known for its broad range of functions, affecting cellular activities such as differentiation, proliferation, and metabolism 64-68. c-Myc functions as an oncogene in numerous cancers, including OSCC 69, 70.

## Conclusion and Future Perspectives

In recent years, significant advancements in next-generation sequencing technology have led to the identification of numerous tumor-related lncRNAs 71-74. These lncRNAs have emerged as potential oncogenes or tumor suppressor genes, playing crucial regulatory roles in tumor development processes 11, 75-77. The extensive body of evidence highlights the importance of lncRNAs in unraveling the intricate mechanisms underlying cancer progression.

One such lncRNA, HOXB-AS3, has garnered significant attention in the field of tumorigenesis recently. LncRNA HOXB-AS3 has been found to be upregulated in various human cancers, including solid and hematological tumors 41, 43, 45, 48, 50. And lncRNA HOXB-AS3 levels have shown significant correlations with some clinicopathological features and prognosis, such as TNM stage, tumor metastasis status, and overall survival. In-depth analyses using data from TCGA and UCSC XENA databases further indicate that lncRNA HOXB-AS3 holds promise as both a prognostic and diagnostic biomarker for diverse malignancies.

Moreover, HOXB-AS3 has emerged as a promising therapeutic target in cancer treatment. This lncRNA engages in diverse interactions with molecules, functioning as a multifaceted scaffold that intricately regulates gene expression and cellular dynamics. It plays a role in regulating gene expression at the transcriptional level through interactions with key proteins such as DNMT1 47, EZH2 49, and PTBP1 48. Additionally, lncRNA HOXB-AS3 contribute to tumor progression by participating in several signaling pathways, including p53 signaling 47, PI3K-AKT pathway 43, Wnt/β-catenin signaling 44, and lipid metabolism pathways 48, underscoring its pivotal role in maintaining cellular equilibrium and influencing tumor growth. And HOXB-AS3's involvement extends to crucial tumor-related processes like cell proliferation, migration, and invasion. Targeting its expression through methods like small interfering RNA (siRNA) or small hairpin RNA (shRNA) has yielded encouraging outcomes. For instance, siRNA-mediated interference in hepatoma cells curtails proliferation, induces apoptosis, and leads to cell cycle arrest 47. In ovarian cancer 41, 44, suppressing HOXB-AS3 hampers cell proliferation and impedes migration and invasion. Similar siRNA-based approaches in endometrial carcinoma 42 and acute myeloid leukemia 50 demonstrate reduced cancer cell proliferation and enhanced apoptosis. Moreover, in lung cancer 43, shRNA-mediated inhibition of HOXB-AS3 curbs cell proliferation, migration, and invasion, triggers apoptosis, and diminishes tumorigenicity in mouse models. These findings collectively suggest that HOXB-AS3 may be a viable target for drug development. Additionally, HOXB-AS3 has been identified as a significant player in sorafenib resistance 49, cancer stemness 49, 50, and immune escape 50, offering new therapeutic avenues for cancer patients.

Intriguingly, although initially categorized as a lncRNA, debates have arisen regarding HOXB-AS3's potential translation into a micro-peptide 40, 52. Reports have presented varying effects, suggesting oncogenic and tumor-suppressive roles in colon 40 and oral cancers 52, respectively. Additionally, lower levels of the HOXB-AS3 peptide have been linked to more advanced clinical stages and a poorer prognosis for patients with CRC 40. These findings raise the possibility that the HOXB-AS3 peptide could serve as a prognostic biomarker and therapeutic target in certain tumor types.

Despite the progress in HOXB-AS3 research, the field is still fraught with challenges and open questions. A primary uncertainty is whether HOXB-AS3 encodes micro-peptides across various human tumors and the extent of aberrant HOXB-AS3 micro-peptide expression, including their potential prognostic and diagnostic implications for diverse tumor types. Addressing these gaps necessitates further study. Moreover, it is vital to unravel the specific mechanisms through which HOXB-AS3 operates in different tumor contexts, whether via RNA-mediated pathways or through the actions of its encoded proteins. Thorough validation with *in vitro* and *in vivo* studies is essential to establish definitive evidence. Secondly, the utility of HOXB-AS3 as a diagnostic marker remain to be further explored. Our review suggests that HOXB-AS3 expression in tissues may serve as a promising diagnostic predictor to distinguish between tumor and normal samples across a range of malignancies. Nonetheless, the verification of the ROC curve is preliminary and based on the available dataset. It is required for broader clinical investigations for validation. To date, the detection of HOXB-AS3 in body fluids like plasma, and its feasibility as a non-invasive, and cost-effective tumor detection method remains unreported. However, the literature does report other lncRNAs, such as LINK-A 78-81 and HOTAIR 82-84, which are found to be significantly expressed in the blood of patients with diverse tumors and proposed as a non-invasive diagnostic marker with substantial diagnostic potential in diverse tumorigenic conditions. This highlights the imperative for increased focus and research into the potential of HOXB-AS3 as a tumor biomarker in these readily accessible biofluids. Lastly, the prognostic value of HOXB-AS3 is currently grounded in retrospective clinical samples. Prospective studies or clinical trials, notably those that are large-scale and multi-center, encompassing diverse patient groups, are crucial to delve deeper into the prognostic significance of HOXB-AS3. These future research efforts will be instrumental in enriching our collective understanding of HOXB-AS3 and its clinical relevance in oncology.

In conclusion, HOXB-AS3 plays a multifaceted role in tumorigenesis and tumor progression, potentially serving as a cancer-specific biomarker for diagnosis, prognosis, and treatment. Its involvement in tumorigenesis is complex, encompassing both RNA and protein levels. The recent discovery of its translation into a micro-peptide adds a new dimension to its functional characterization. Future research should aim to resolve the ongoing debate regarding HOXB-AS3's RNA and protein functions and their respective contributions to tumorigenesis. Comprehensive studies investigating the interplay between HOXB-AS3 RNA and protein levels will provide valuable insights into its therapeutic potential as a cancer treatment target.

## Supplementary Material

Supplementary table.

## Figures and Tables

**Table 1:** Expression of HOXB-AS3 in tissues and cells and its relationship with clinical characteristics and survival in malignancies.

Tumor type	RNA or protein	Expression level	Models			Clinical characteristic	Survival indicator	Prognosis with high expression	Biomarker	Ref.
			Tissue	Cell line	Main subcellular location					
Hepatoma	RNA	Up	Human tissues	HepG, PLC, Hep3B, LM3	Nucleus (Hep3B cells)	TNM stage	-	-	-	47
Liver cancer	RNA	Up	Human tissues	-	-	-	-	-	-	49
Acute myeloid leukemia	RNA	Up	Human tissues, TCGA	OCI/AML3, TF-1	Cytoplasm (OCI/AML3 cells)	Sex, age, PLT, cytogenetic risk group S, relapse rate, somatic gene mutations	Overall survival, Relapse free survival (human)	Adverse	Prognostic	46
NPM1-mutated acute myeloid leukemia	RNA	Up	Human tissues	Kasumi-1, KG-1a, K-562, MV-4-11, THP-1, MOLM-13, OCI-AML3	Nuclear (OCI-AML3 cells)	-	Overall survival (murine PDX model)	Adverse	Prognostic	45
Acute myeloid leukemia	RNA	Up	Human tissues, GSE dataset, TARGET, TCGA	HS-5, THP-1, leukemic stem cells (isolated from THP-1 cells)	-	-	Overall survival (human and murine model)	Adverse	Prognostic	50
Epithelial ovarian cancer	RNA	Up	Patient specimens, TCGA, GEPIA	HEY, SKOV3, OVCAR3, IOSE80	-	Histological grade, FIGO stage, lymph node metastasis	Overall survival (human)	Adverse	Prognostic	44
Epithelial ovarian cancer	RNA	Up	Human sample, TCGA	-	-	Disease-free status and overall survival status	Disease-free survival, Overall survival (human)	Adverse	Prognostic	41
Lung cancer	RNA	Up	Human sample	H1795, SPC-A1, H460, A549, 16HBE	-	Differentiation, TNM	-	-	-	43
Endometrial carcinoma	RNA	Up	Human sample, TCGA	HEC-1-A, HEC-1-B, Ishikawa, ESC	-	-	Overall survival (human)	-	Prognostic	42
Uterine corpus endometrial carcinoma	RNA	Up	-	hEM15A, HEC1A,	-	-	-	-	-	51
Endometrioid carcinoma	RNA	Up	Human tissues, GSE dataset, TCGA	Ishikawa, AN3-CA, HEC-1A, RL95-2	-	-	-	-	Diagnostic	48
Colon cancer	RNA	Down	Human sample, GEO dataset	SW480, SW620, HCT116, HCT116^high^	-	-	-	-	-	40
	Micro‑peptide	Down	Human sample	SW480, SW620, HCT116, HCT116^high^	Nucleus (SW480, SW620)	Clinical stage	Overall survival (human)	Favorable	Prognostic	
Oral squamous cell carcinoma	RNA	Up	Human sample, TCGA	-	-	TNM stage	Overall survival (human)	Adverse	Prognostic	52
	Micro‑peptide	Up	Human sample	-	-	-	-	-	-	

**Table 2:** The role and regulatory mechanism of HOXB-AS3 RNA and micro-peptide in human malignancies.

Level	Cancer type	Role	Experiments	*In vitro*	*In vivo*	Functions	Related molecule/signal	Ref.
RNA	Hepatoma	Oncogenic	*In vitro*	Hep3B, LM3	-	Cell proliferation, apoptosis, cell cycle	p53, DNMT1	47
	Liver cancer	Oncogenic	*In vitro* and *in vivo*	Huh7, Huh7/SR	xenograft mouse model (SCID mice)	Sorafenib resistance, cancer stemness	EZH2, Dicer, H3K27me3, SOX2, OCT4	49
	Acute myeloid leukemia	Oncogenic	*In vitro*	OCI/AML3, TF-1	-	Cell proliferation	CDK1, CCNB2, CDC25A, PCNA, CDC6, MCM4, MCM6	46
	NPM1-mutated acute myeloid leukemia	Oncogenic	*In vitro* and *in vivo*	Leukemic stem cells	Human AML patient-derived xenografts (NSG mice)	Proliferation	EBP1, NPM1	45
	Acute myeloid leukemia	Oncogenic	*In vitro* and *in vivo*	THP-1 cell, leukemic stem cells	Xenograft mouse model (NSG mice)	Proliferation, apoptosis, immune escape	YTHDC1, NR_033205.1	50
	Epithelial ovarian cancer	Oncogenic	*In vitro*	SKOV-3, OVCAR-3	-	Cell proliferation, migration, invasion, EMT, apoptosis	β-catenin, cyclin D1, c-Myc, Wnt/β-catenin signaling	44
	Epithelial ovarian cancer	Oncogenic	*In vitro*	SKOV3, A2780	-	Proliferation, invasion, migration	LDHA, miR-378a-3p	41
	Lung cancer	Oncogenic	*In vitro* and *in vivo*	A549, H1975	Xenograft mouse model (BALB/c nude mice)	Cell proliferation, apoptosis, cell cycle, migration, invasion, tumor growth	p-AKT, pI3Kp85a, PI3K/AKT pathway	43
	Endometrial carcinoma	Oncogenic	*In vitro*	HEC1A, Ishikawa cells	-	Cell proliferation, apoptosis	ADAM9, miR-498-5p	42
	Uterine corpus endometrial carcinoma	Oncogenic	*In vitro*	HEC1A cells	-	Proliferation, migration	-	51
	Endometrioid carcinoma	Oncogenic	*In vitro*	Ishikawa, AN3-CA, HEC-1A, RL95-2	-	Proliferation, invasion, cell cycle, apoptosis, lipid metabolism	PTBP1, SREBP1	48
Micro-peptide	Colon cancer	Anti-oncogenic	*In vitro* and *in vivo*	SW480, HTC-116, SW620	Xenograft mouse model (NOD-SCID mice)	Colony formation, tumor growth, migration, invasion, metastasis, metabolic reprogramming	hnRNPA1, PKM2, PKM1	40
	Oral squamous cell carcinoma	Oncogenic	*In vitro*	Cal-27, UM2	-	Proliferation, viability	IGF2BP2, c-Myc	52

## Abbreviations

AML: Acute myeloid leukemia
AUC: Area under the curve
CRC: Colorectal cancer
DFI: Disease-free interval
DSS: Disease-specific survival
EBP1: ErbB3-binding protein 1
EC: Endometrial carcinoma
EMT: Epithelial-mesenchymal transition
HOXB-AS3: HOXB Cluster Antisense RNA 3
LncRNAs: Long non-coding RNAs
LSCs: Leukemic stem cells
OS: Overall survival
ROC: Receiver operating characteristic
TCGA: The Cancer Genome Atlas
