# Supplementary material for: A concise review of the regulatory, diagnostic, and prognostic implications of HOXB-AS3 in tumors
Source: J Cancer. 2024 Jan 1;15(3):714–28. doi: 10.7150/jca.91033 (PMC10777036; doi:10.7150/jca.91033)
Supplement: Supplementary file 1 — Supplementary table. [file jcav15p0714s1.pdf]

Supplementary Table 1. Abbreviations in Figures 2, 3, 4 and 5.

Abbreviation	Full name
**ACC**	Adrenocortical carcinoma
**BLCA**	Bladder Urothelial Carcinoma
**BRCA**	Breast invasive carcinoma
**CESC**	Cervical squamous cell carcinoma and endocervical adenocarcinoma
**CHOL**	Cholangio carcinoma
**COAD**	Colon adenocarcinoma
**DLBC**	Lymphoid Neoplasm Diffuse Large B-cell Lymphoma
**ESCA**	Esophageal carcinoma
**ESAD**	Esophageal adenocarcinoma
**ESCC**	Esophageal squamous cell carcinoma
**GBM**	Glioblastoma multiforme
**HNSC**	Head and Neck squamous cell carcinoma
**KICH**	Kidney Chromophobe
**KIRC**	Kidney renal clear cell carcinoma
**KIRP**	Kidney renal papillary cell carcinoma
**LAML**	Acute Myeloid Leukemia
**LGG**	Brain Lower Grade Glioma
**LIHC**	Liver hepatocellular carcinoma
**LUAD**	Lung adenocarcinoma
**LUSC**	Lung squamous cell carcinoma
**MESO**	Mesothelioma
**OSCC**	Oral squamous cell carcinoma
**OV**	Ovarian serous cystadenocarcinoma
**PAAD**	Pancreatic adenocarcinoma
**PCPG**	Pheochromocytoma and Paraganglioma
**PRAD**	Prostate adenocarcinoma
**READ**	Rectum adenocarcinoma
**SARC**	Sarcoma
**SKCM**	Skin Cutaneous Melanoma
**STAD**	Stomach adenocarcinoma
**TGCT**	Testicular Germ Cell Tumors
**THCA**	Thyroid carcinoma
**THYM**	Thymoma
**UCEC**	Uterine Corpus Endometrial Carcinoma
**UCS**	Uterine Carcinosarcoma
**UVM**	Uveal Melanoma
